# *Plasmodium chabaudi* AS Infection Induces CD4^+^ Th1 Cells and Foxp3^+^T-bet^+^ Regulatory T Cells That Express CXCR3 and Migrate to CXCR3 Ligands

**DOI:** 10.3389/fimmu.2019.00425

**Published:** 2019-03-11

**Authors:** Floriana Berretta, Ciriaco A. Piccirillo, Mary M. Stevenson

**Affiliations:** ^1^Research Institute of the McGill University Health Centre, Montreal, QC, Canada; ^2^Centre for Host-Parasite Interactions, Institute of Parasitology, McGill University, Ste-Anne de Bellevue, QC, Canada; ^3^Department of Microbiology and Immunology, McGill University, Montreal, QC, Canada; ^4^Program in Infectious Disease and Immunity in Global Health and Centre of Excellence in Translational Immunology, Research Institute of the McGill University Health Centre, Montreal, QC, Canada

**Keywords:** malaria, CXCR3, CD4^+^ T cells, regulatory T cells, T-bet

## Abstract

Control and elimination of blood-stage *Plasmodium chabaudi* AS infection requires CD4^+^ Th1 cells that secrete IFN-γ and T follicular help (Tfh) cells together with B cell production of antibody. Foxp3^+^ regulatory T cells (Tregs) are also crucial to protect the host from immunopathology and severe disease, but these cells can suppress protective immune responses to malaria. The chemokine receptor CXCR3 expressed by activated T cells is important for trafficking of CD4^+^ Th1 cells to sites of inflammation and infection. Previous studies demonstrated CXCR3 is expressed on CD4^+^ T cells in the spleen during malaria, but the phenotype was not defined. We identified the phenotype of CD4^+^ T cells that expressed CXCR3 in C57BL/6 (B6) mice during acute *P. chabaudi* AS infection by analyzing expression of the transcription factors T-bet and Foxp3. We also investigated if CXCR3 contributes to control of parasite replication and survival. The frequency and number of CD4^+^CXCR3^+^ T cells increased dramatically in the spleen of infected B6 mice coincident with increased CD4^+^IFN-γ^+^ T cells. CXCR3 was up-regulated on effector CD4^+^Foxp3^−^ T cells as well as Foxp3^+^ Tregs. Consistent with our previous observations, CD4^+^T-bet^+^Foxp3^−^ T cells increased in B6 mice during acute infection. T-bet^+^Foxp3^+^ Tregs also increased significantly and a high frequency of these cells expressed CXCR3 supporting the notion that these cells may be Th1-like Tregs. Despite this, the percentage of CD4^+^Foxp3^+^ Tregs from infected B6 mice that migrated *in vitro* to the CXCR3 ligands CXCL9 and CXCL10 was significantly less than naïve mice. To investigate the *in vivo* contribution of CXCR3 to control of acute blood-stage malaria, we compared the course and outcome of *P. chabaudi* AS infection in wild-type (WT) B6 and CXCR3-deficient mice. Parasitemia levels were significantly higher around the time of peak parasitemia in CXCR3^−/−^ compared to WT mice but survival was similar suggesting a role for CXCR3 in controlling parasite replication during acute *P. chabaudi* AS infection. Together, our findings indicate Th1-like CD4^+^T-bet^+^Foxp3^+^ Tregs that express CXCR3 are induced during acute blood-stage malaria and suggest CXCR3 expression on CD4^+^ Th1 cells may contribute to their migration to the spleen.

## Introduction

Malaria remains a major global health threat with 90% of the disease burden in sub-Saharan Africa (1). In 2016, there were 216 million cases of malaria world-wide which represents an increase of 5 million cases compared to 2015 even though the number of deaths remained at ~445,000 per year. Despite extensive studies in humans and mice infected with *Plasmodium* to identify the immune mechanisms required for protection against blood-stage infection, important gaps in our knowledge remain. CD4^+^ T cell-B cell interactions are essential for control of parasite replication and elimination of infection (2). CD4^+^ Th1 cells that express T-bet and secrete IFN-γ and T follicular helper (Tfh) cells, crucial for generating antibody-mediated immunity, play important roles (2). Immunoregulatory mechanisms including the anti-inflammatory cytokine IL-10 and regulatory T cells (Tregs) are vital to protect the host from immunopathology and severe disease (3–5). On the other hand, such mechanisms may suppress protective immune responses.

Although earlier studies on the role of CD4^+^Foxp3^+^ Tregs in immunity to malaria were not conclusive, recent findings in mice infected with *Plasmodium chabaudi* AS or *P. yoelii* support the notion that Tregs suppress Th1 as well as Tfh cell responses (4, 6, 7). Indeed, higher blood parasitemia levels are associated with higher frequencies of CD4^+^Foxp3^+^ Tregs in humans and mice with malaria. Conversely, a lower frequency of Tregs is associated with better disease outcome. During the acute phase of *P. chabaudi* AS, C57BL/6 (B6) mice, used in the present study, have a significant increase in effector CD4^+^ Th1 cells that express T-bet and secrete IFN-γ (6, 8). The accumulation and expansion of CD4^+^ Th1 cells that secrete IFN-γ in the spleen are essential for control and elimination of *P. chabaudi* AS infection (9). Although CD4^+^Foxp3^+^ Tregs increase significantly in infected compared to naïve B6 mice, there is a high ratio of effector CD4^+^ T cells to Tregs in these hosts during acute *P. chabaudi* AS infection (6).

The lymphocyte-specific chemokine receptor CXCR3 expressed by activated T cells as well as NK cells is important for CD4^+^ Th1 cell migration to sites of inflammation and infection (10, 11). Interaction of CXCR3 with its ligands, the C-X-C chemokines CXCL9 (monokine induced by IFN-γ), CXCL10 (interferon-induced protein-10), and CXCL11 (interferon-inducible T-cell alpha chemoattractant) contributes to Th1 cell differentiation (12). The C-X-C chemokines are induced by IFN-γ and are produced by several immune cells including macrophages and dendritic cells as well as non-immune cells.

CXCR3 and other chemokine receptors have been demonstrated to be up-regulated in CM patients and during ECM in mice (13–16). Susceptibility to ECM in *P. berghei* ANKA-infected B6 mice requires CXCR3 expression on pathogenic CD8^+^ T cells (17). *Cxcr3*^−/^^−^ mice are protected from ECM due to reduced CD8^+^ T cell sequestration in the brain. Interestingly, CXCR3 expression is also highly up-regulated on CD4^+^ and CD8^+^ T cells in the spleen during *P. berghei* ANKA infection (15). However, neither the role of CXCR3 expression on T cells in the spleen during malaria nor the phenotype of the T cells, especially of CD4^+^ T cells, expressing this chemokine receptor have been investigated in mice infected with *P. berghei* ANKA or in other rodent malaria models. In the present study, we examined CXCR3 expression on CD4^+^ T cells in the spleen of B6 mice infected with *P. chabaudi* AS and identified the CD4^+^ T cell population that express it.

The T-box transcription factor T-bet is essential for Th1 cell differentiation and effector function due to its ability to activate transcription of the IFN-γ gene (18). Interestingly, T-bet is expressed on a subset of CD4^+^Foxp3^+^ T cells and is thought to be involved in the homeostasis and function of these cells during Th1 inflammation (19). Recently, it was observed that Foxp3^+^ Tregs that express T-bet increase in individuals with acute *P. vivax* infection (20). In addition to activating transcription of the IFN-γ gene, T-bet induces the transcription of other Th1-associated genes including *Cxcr3* inducing up-regulation of CXCR3 expression on effector CD4^+^ T cells (21). Increased CXCR3 expression on CD4^+^Foxp3^+^ T cells likewise requires T-bet and occurs via a IFN-γ-dependent mechanism (19).

Given the complex interactions between CXCR3 and the transcription factor T-bet on effector CD4^+^Foxp3^−^ T cells and CD4^+^Foxp3^+^ Tregs, we questioned if these Th1-associated markers are differentially expressed on CD4^+^ T cell populations during acute *P. chabaudi* AS infection in B6 mice. To address this, we investigated if CXCR3 expression is up-regulated on CD4^+^ T cell populations in the spleen of infected mice. We also determined if CXCR3 and T-bet are co-expressed on effector CD4^+^Foxp3^−^ T cells and CD4^+^Foxp3^+^ Tregs during infection. Because we observed that C-X-C chemokine expression is up-regulated in the spleen during *P. chabaudi* AS infection, we performed *in vitro* migration assays to study the chemotactic ability of CD4^+^ T cells from infected mice to CXCL9, CXCL10, and CXCL11. Finally, we investigated the *in vivo* role of CXCR3 to immunity to blood-stage malaria by determining the course and outcome of *P. chabaudi* AS infection in wild-type (WT) B6 compared to CXCR3-deficient mice.

## Materials and Methods

### Mice and Parasites

WT B6 mice were purchased from Charles River Laboratories (St. Constant, QC). B6.129P2-Cxcr3^tm1Dgen^/J (CXCR3^−/−^) mice were obtained from Jackson Laboratories (Bar Harbor, ME). Mice were maintained in the animal facility of the Research Institute of the McGill University Health Center (Montreal, QC) under specific-pathogen-free conditions. Female mice, 6–8 weeks old, were used for all experiments. Experiments were conducted using procedures approved by the Canadian Council on Animal Care and the Animal Care Committee of the Research Institute of the McGill University Health Center. Blood-stage *P. chabaudi* AS parasite was maintained in mice by weekly passage as previously described (22). Mice were infected by intraperitoneal (i.p.) injection of 1 × 10^6^ parasitized red blood cells (pRBC). Parasitemia was monitored in the blood by microscopic examination of Diff-Quick (Fisher Scientific)-stained blood smears.

### Cell Preparation

Spleens from naïve and *P. chabaudi* AS infected B6 mice were collected aseptically at the indicated times post-infection (p.i.). To prepare single cell suspensions, tissues were perfused with PBS containing 1% FCS (HyClone Laboratories, Logan, UT), teased apart, and gently pressed through a sterile fine wire mesh. The cells were centrifuged, re-suspended in 0.175 M NH_4_Cl to lyse red blood cells (Sigma-Aldrich, St. Louis, MO), washed, and re-suspended in complete RPMI 1640 medium (Life Technologies, Burlington, ON, Canada) supplemented with 5% heat-inactivated FCS, 25 mM HEPES (Life Technologies), 0.12% gentamicin (Life Technologies), and 2 mM glutamine (Life Technologies). The total number of spleen cells obtained from individual mice was determined using a hemocytometer. Cell viability was determined by trypan blue exclusion (Life Technologies) and was always >95%. For some experiments, CD4^+^ T cells were purified from single cell suspensions prepared from spleens of naïve and infected mice at the indicated times p.i. using a negative CD4^+^ T cell isolation kit (Miltenyi Biotec) following the manufacturer's instructions.

### Immunophenotyping

Single cell suspensions of splenocytes, prepared as described above, were adjusted to 1–2 × 10^6^ cells/ml and stained with viability dye (eFluor® 780 or eFluor® 506; eBioscience, San Diego, CA). Prior to immunophenotyping, Fc receptors were blocked with anti-mouse CD16/CD32 monoclonal antibody (mAb) (clone 2.4G2; BD Biosciences, San Jose, CA) and the cells were surface stained with FITC-conjugated anti-CD4 mAb (clone GK1.5; eBioscience) and PE-conjugated anti-CXCR3 mAb (clone CXCR3-173; eBioscience). For intracellular staining, the cells were surface stained, fixed, and permeabilised using an intracellular fixation kit (eBioscience) according to the manufacturer's instructions, and intracellularly stained with APC-labeled anti-Foxp3 (clone FJK-16s; eBioscience) and anti-T-bet (clone 4B10; eBioscience) mAb. For each antibody, staining was compared to cells stained with an appropriate isotype control antibody. Flow cytometry was performed using a FACSCanto (BD Biosciences) for acquisition, and data were analyzed using FlowJo software (TreeStar).

### Intracellular Cytokine Staining

Purified CD4^+^ T cells obtained from naïve and infected mice were resuspended to 1 × 10^6^ cells/ml in complete RPMI 1640 medium and stimulated with 50 ng/ml PMA and 1 μM ionomycin (Sigma-Aldrich) for 5 h at 37°C and 5% CO_2_ in the presence of 1 μl/ml Brefeldin A (BD Biosciences) to inhibit cytokine secretion. Cells were harvested and surface stained with fluorochrome-conjugated antibodies as described above. After fixation and permeabilization, the cells were stained for intracellular cytokine expression using APC-conjugated, anti-IFN-γ mAb (clone XMG1.2, eBioscience). Cells were also stained for intracellular T-bet expression as described above to identify Th1 cells. Cells were gated on CD4^+^ cells for FACS analysis and data were analyzed using FlowJo software (TreeStar).

### Chemotaxis Assay

For migration studies, 5 × 10^5^ purified CD4^+^ T cells in complete RPMI 1640 medium were added to the top chambers of a 96 well Transwell plate with a pore size of 5 μm (Corning Costar, Acton, MA). Mouse recombinant chemokines CXCL9, CXCL10, CXCL11, and CCL21 (Peprotech, Rocky Hill, NJ) were diluted in complete RPMI 1640 medium and added to the bottom chamber of the Transwell plate to a final concentration of 200 μM for CXCL9 and CXCL11 and 100 μM for CXCL10 and CCL21. As a control, complete RPMI 1640 medium alone was added to the bottom chamber. After incubation for 4 h at 37°C in 5% CO_2_, the cells were collected from the bottom chamber and counted in a hemocytometer. The chemotaxis index was calculated by dividing the number of cells that migrated in response to chemokines by the number of cells in the bottom chamber containing medium alone.

To identify the population of cells that had migrated to each chemokine, cells were collected from individual bottom chambers and surface stained with FITC-conjugated CD4 mAb followed by intracellular staining with APC-conjugated Foxp3 mAb as previously described. Cells were analyzed by FACS and the percentage of CD4^+^Foxp3^+^ Tregs in the migrated cell population was determined.

### RT-PCR

To investigate the expression of chemokine genes, spleens were harvested from naïve and infected B6 mice on the indicated days p.i. and immediately snap-frozen in liquid nitrogen. Frozen tissues were stored at −80°C until processing. Total RNA was extracted and purified using the RT^2^-qPCR-grade mRNA isolation kit (Qiagen) according to the manufacturer's instructions. RNA integrity was verified by denaturing agarose gel electrophoresis, followed by staining with ethidium bromide, and visualized on a UV trans-illuminator. Purity and quantification of RNA were determined by UV absorbance ratio of A260/A280. For first strand cDNA synthesis, 1 μg of purified RNA was used. First strand cDNA synthesis and RT-PCR were performed using reagents and protocols provided by the manufacturer in the RT^2^ Profiler® PCR Kit for Mouse Chemokines and Chemokine Receptors (Qiagen). Reactions were performed in a StepOne® real-time PCR instrument (Applied Biosystems) using a pre-set, standard run thermal cycling condition of 40 cycles. Raw C_t_ data were analyzed using the RT^2^ Profiler® software provided on the manufacturer's web portal. Data are presented as fold increase in mRNA of infected over naïve mice.

### Statistical Analysis

Data are presented as mean ± standard error of the mean (SEM). The statistical significance of differences between groups was analyzed using Kruskal Wallis for one-way analysis of variance. Survival data were analyzed using Martel-Cox log rank test. All statistical analyses were performed using Prism 5 software (GraphPad, San Diego, CA). *p* < 0.05 was considered significant.

## Results

### CXCR3 Expression Increases on Splenic CD4^+^ T Cells During Acute *P. chabaudi* AS Infection and Is Expressed on Both Effector CD4^+^Foxp3^−^ and CD4^+^Foxp3^+^ Regulatory T Cells

**Figure 1:**
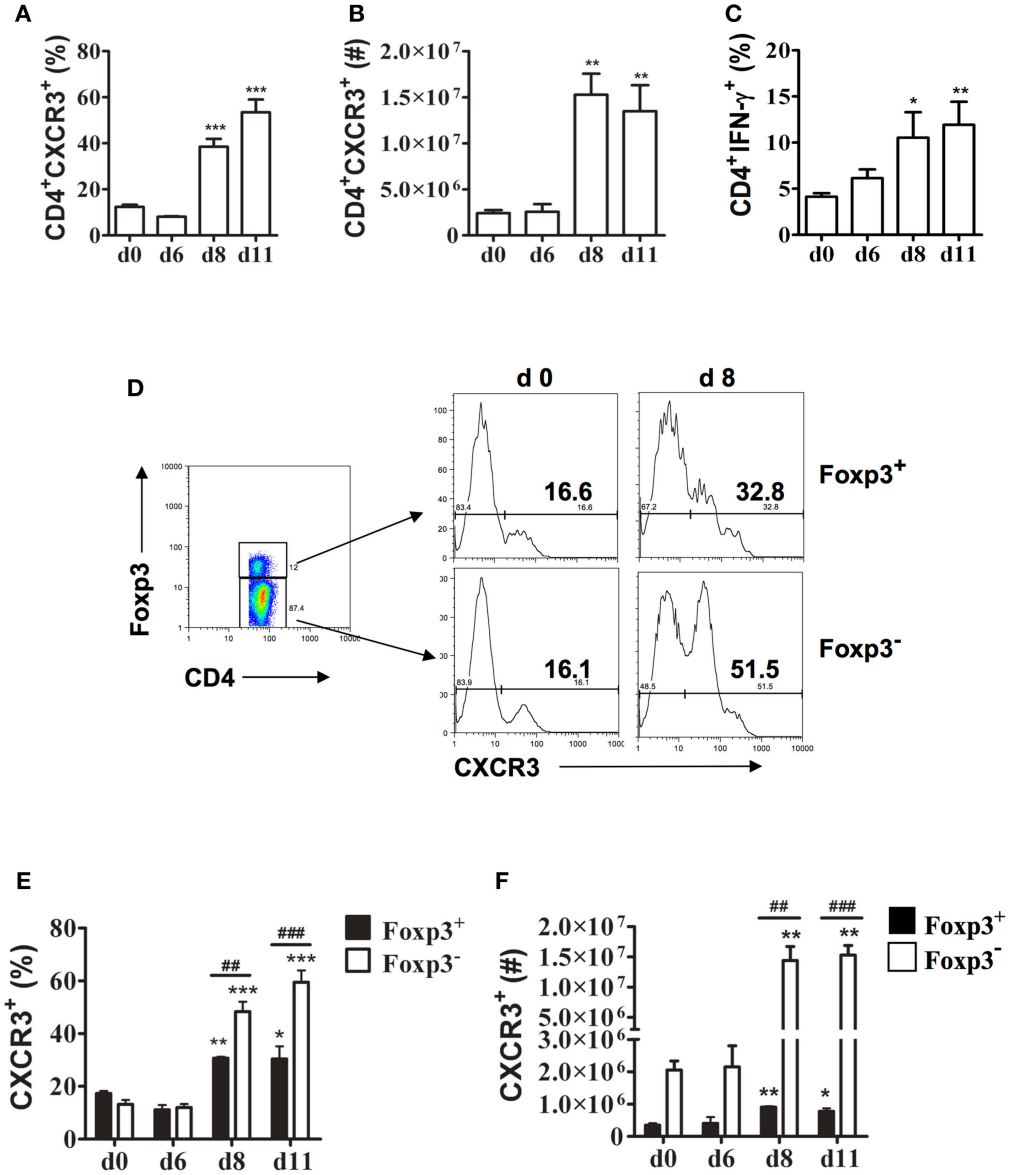
CXCR3 expression is up-regulated on splenic CD4^+^Foxp3^−^ and CD4^+^Foxp3^+^ T cells during *P. chabaudi* AS infection. Spleen cells were collected from naïve (d0) and infected B6 mice on the indicated days after i.p. infection with 1 × 10^6^ *P. chabaudi* AS pRBC. **(A,B)** The cells were stained with FITC-conjugated anti-CD4 and PE-conjugated anti-CXCR3 mAbs and analyzed by flow cytometry. The frequency **(A)** and number **(B)** of CD4^+^CXCR3^+^ cells are shown. Data are presented as mean ± SEM and are representative of one of two replicate experiments with 3–5 mice at each time point. ^**^p < 0.01; ^***^p < 0.005. **(C)** CD4^+^ T cells were purified from the spleens of naïve and infected B6 mice on the indicated days p.i. and stimulated with 50 ng/ml PMA and 1 μM ionomycin for 5 h in the presence of 1 μl/ml Brefeldin A. Cells were harvested and surface stained with FITC-conjugated anti-CD4 mAb, fixed and permeabilised, and intracellularly stained with APC-conjugated anti-IFN-γ-mAb. Gated CD4^+^ cells were analyzed by flow cytometry for the frequency of IFN-γ^+^ cells. Data are presented as mean ± SEM and are representative of one of two replicate experiments with 3–5 mice at each time point. ^*^p < 0.05; ^**^p < 0.01. **(D–F)** Spleen cells from naïve (d0) and infected WT mice were collected and surface stained with fluorochrome-conjugated anti-CD4 and anti-CXCR3 mAbs and intracellularly stained with APC-labeled anti-Foxp3 mAb. The gating strategy used for FACS analysis and representative plots from a naïve (d0) mouse and a mouse on day 8 p.i. are shown **(D)**. The frequency **(E)** and number **(F)** of CXCR3^+^ cells within the CD4^+^Foxp3^−^ and CD4^+^Foxp3^+^ populations at various times p.i. are shown. Data are presented as mean ± SEM and are representative of one of two replicate experiments with 3–5 mice at each time point. ^*^p < 0.05; ^**^p < 0.01; and ^***^p < 0.005 for Foxp3^−^ and Foxp3^+^ cells from infected compared to naïve (d0) mice. ^*##*^*p* < 0.01; ^*###*^*p* < 0.005 for Foxp3^−^ compared to Foxp3^+^ cells.

Previous studies showed that CXCR3 expression increases on CD4^+^ and CD8^+^ T cells in the spleen during *P. berghei* ANKA infection suggesting this chemokine receptor may be important in T cell trafficking to the spleen during malaria (15). During *P. chabaudi* AS infection, the spleen is the major site of accumulation of CD4^+^ T cells especially CD4^+^T-bet^+^IFN-γ^+^ Th1 cells that are essential to control parasitemia during the first 2 weeks after infection (6, 23). To further investigate the role of CXCR3 in *P. chabaudi* AS infection, we determined CXCR3 expression on CD4^+^ T cells in the spleen of infected B6 mice using the gating strategy shown in Supplementary Figure 1A. The frequencies and numbers of CD4^+^CXCR3^+^ T cells increased significantly in the spleen of *P chabaudi* AS-infected compared to naïve B6 mice on days 8 and 11 p.i. (Figures 1A,B). Importantly, the increases in CD4^+^CXCR3^+^ T cells coincided with significant increases in CD4^+^IFN-γ^+^ T cells suggesting the importance of CXCR3 in the accumulation of effector Th1 cells in the spleen during acute *P. chabaudi AS* infection (Figure 1C).

Previously, we observed that CD4^+^Foxp3^+^ Tregs increase significantly in the spleen of infected B6 mice during acute *P. chabaudi* AS infection followed by a significant increase in effector CD4^+^T-bet^+^IFN-γ^+^ Th1 cells around the time of peak parasitemia (6). Immunohistochemical staining revealed that CD4^+^Foxp3^+^ Tregs are located almost exclusively in the T cell areas of the white pulp within 6 days p.i. and are still evident on day 10 p.i. To address if CXCR3 is expressed differentially on CD4^+^ T cell populations during blood-stage malaria, we examined CXCR3 expression on CD4^+^Foxp3^−^ and CD4^+^Foxp3^+^ T cells (Supplementary Figure 1A). A similarly low frequency of CD4^+^ T cells from naïve B6 mice expressed CXCR3 in gated CD4^+^Foxp3^−^ and CD4^+^Foxp3^+^ T cells (Figure 1D). The frequencies of cells expressing CXCR3 increased among effector CD4^+^Foxp3^−^ T cells as well as CD4^+^Foxp3^+^ Tregs as the infection progressed, with ~50% of effector CD4^+^Foxp3^−^ T cells expressing CXCR3 compared to 33% of CD4^+^Foxp3^+^ Tregs on day 8 p.i. The level of CXCR3 expression was up-regulated on CD4^+^Foxp3^+^ T cells from infected compared to naïve B6 mice as shown by significantly higher MFIs on days 8 and 11 p.i. (Supplementary Figure 1B). Although there were significant increases in the frequencies and numbers of CD4^+^Foxp3^−^ as well as CD4^+^Foxp3^+^ T cells expressing CXCR3 on days 8 and 11 p.i. compared to naïve B6 mice, the increases were significantly greater in effector CD4^+^ T cells compared to CD4^+^Foxp3^+^ Tregs (Figures 1E,F).

### T-bet and CXCR3 Expression Are Up-Regulated on Effector CD4^+^Foxp3^−^ and CD4^+^Foxp3^+^ Regulatory T Cells During Blood-Stage *P. chabaudi* AS

**Figure 2:**
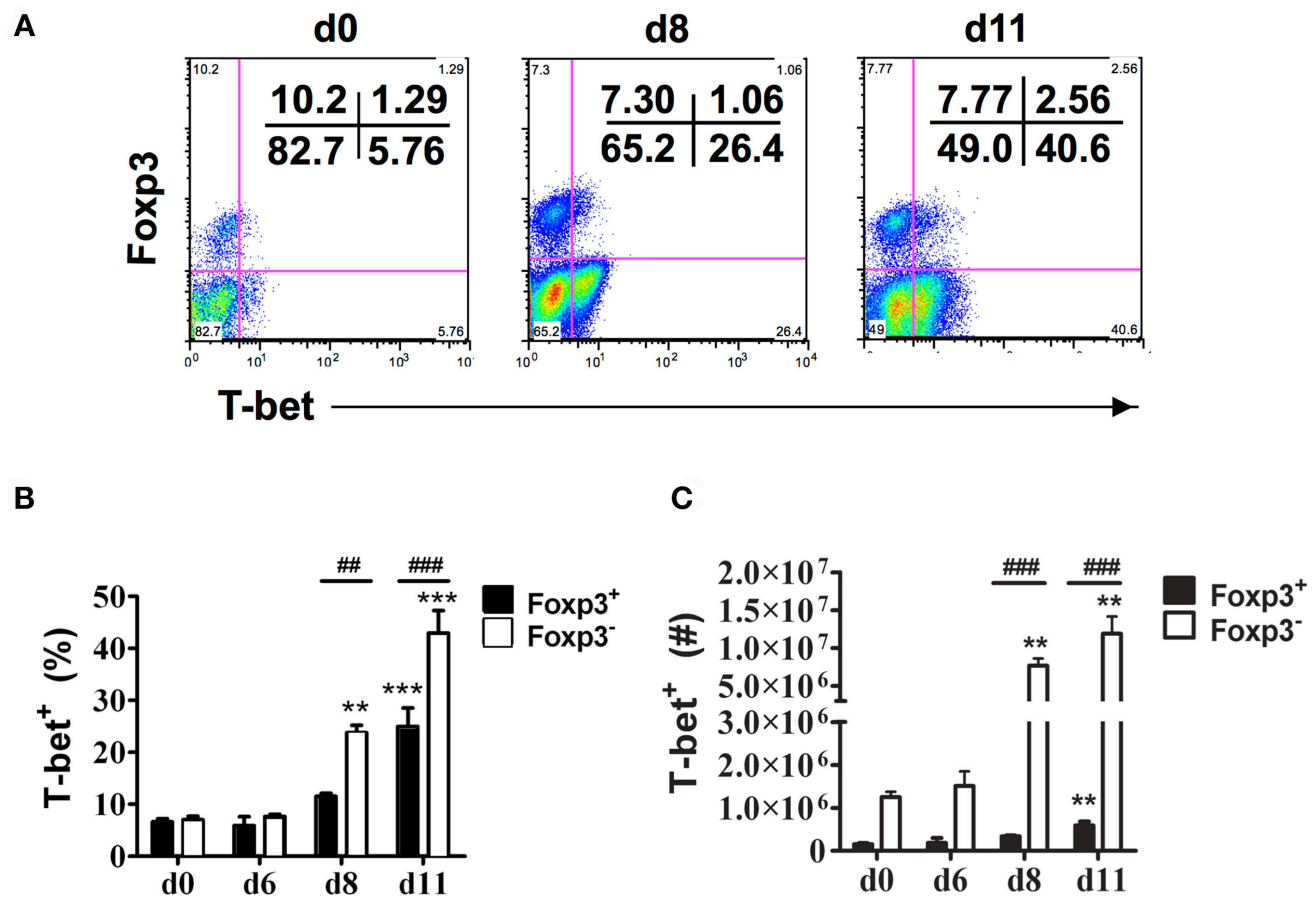
T-bet expression is up-regulated on both effector CD4^+^ T cells and CD4^+^Foxp3^+^ Tregs. Spleen cells were collected from naïve (d0) and infected B6 mice on the indicated days after i.p. infection with 1 × 10^6^ *P. chabaudi* AS pRBC. The cells were surface stained with FITC-conjugated anti-CD4^+^ mAb and intracellularly stained with fluorochrome-conjugated anti-Foxp3 and anti-T-bet mAbs. Gated CD4^+^ T cells were analyzed by flow cytometry for expression of Foxp3 and T-bet. **(A)** Representative dot plots from a naïve (d0) mouse and a mouse on day 8 and day 11 p.i. showing the frequency of T-bet expression in the CD4^+^Foxp3^−^ and CD4^+^Foxp3^+^ populations. The frequency **(B)** and number **(C)** of T-bet^+^ cells in the CD4^+^Foxp3^−^ and CD4^+^Foxp3^+^ populations at various times p.i. are shown. Date are presented as mean ± SEM and are representative of one of two replicate experiments with 3–5 mice at each time point. ^**^p < 0.01; ^***^p < 0.005 for Foxp3^−^ and Foxp3^+^ cells from infected compared to naïve (d0) mice at each time point. ^*##*^*p* < 0.01; ^*###*^*p* < 0.005 for Foxp3^−^ compared to Foxp3^+^ cells.

Consistent with our previous observations, CD4^+^Foxp3^−^ T cells expressing T-bet increased dramatically in the spleen of infected B6 mice on days 8 and 11 p.i. (Figure 2A) (6). The frequency and number of CD4^+^Foxp3^+^ cells expressing T-bet also increased during infection albeit to lower levels than effector CD4^+^ T cells expressing T-bet. On days 8 and 11 p.i., the frequencies and numbers of CD4^+^T-bet^+^Foxp3^−^ T cells were significantly higher in infected compared to naïve B6 mice, while significant increases in frequency and number of CD4^+^T-bet^+^Foxp3^+^ cells were evident only on day 11 p.i. (Figures 2B,C). Notably, the frequency and number of CD4^+^T-bet^+^Foxp3^+^ cells were significantly less than the frequency and number of CD4^+^T-bet^+^Foxp3^−^ T cells on days 8 and 11 p.i.

**Figure 3:**
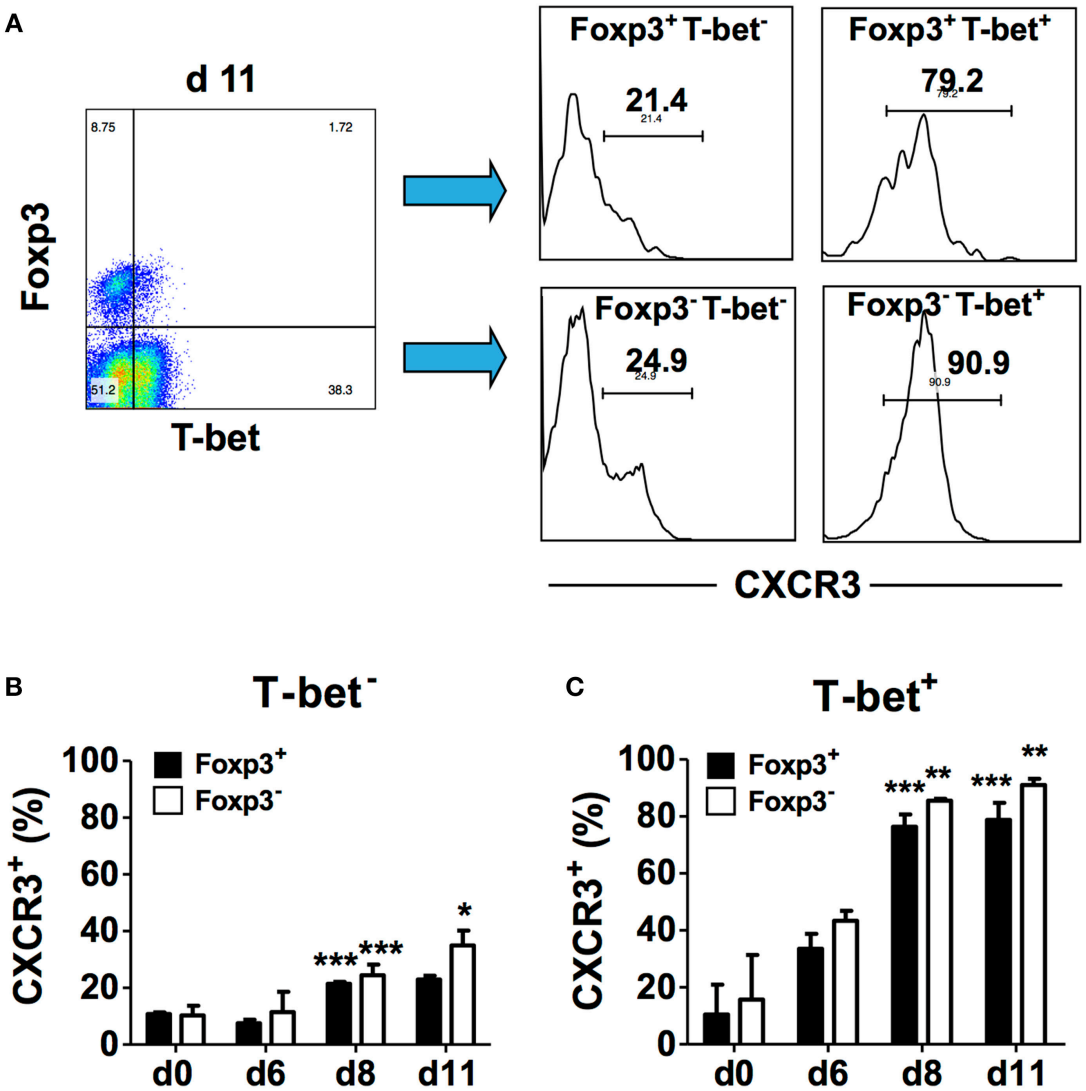
T-bet expression is up-regulated on CD4^+^CXCR3^+^ T cells regardless of Foxp3 expression. Spleen cells were collected from naïve (d0) and infected B6 mice on the indicated days after i.p. infection with 1 × 10^6^ *P. chabaudi* AS pRBC. The cells were surface stained with FITC-conjugated anti-CD4^+^ and PE-conjugated anti-CXCR3 mAbs followed by intracellular staining with fluorochrome-conjugated anti-T-bet and anti-Foxp3 mAbs. The cells were gated on CD4^+^ cells and analyzed for expression of T-bet, Foxp3, and CXCR3. **(A)** Representative plots from one infected mouse showing the gating strategy used to analyze CXCR3 expression within the different CD4^+^ populations. **(B)** The frequency of CXCR3^+^ cells within the Foxp3^+^T-bet^−^ and Foxp3^−^T-bet^−^ populations at various times p.i. **(C)** The frequency of CXCR3^+^ cells within the Foxp3^+^T-bet^+^ and Foxp3^−^T-bet^+^ populations at various times p.i. Data are presented as mean ± SEM and are representative of one of two replicate experiments with 3–5 mice at each time point. ^*^p < 0.05; ^**^p < 0.01; ^***^p < 0.005 for CXCR3 expression on Foxp3^−^ compared to Foxp3^+^ cells.

In addition to activating transcription of the IFN-γ gene, T-bet also induces the transcription of other Th1-associated genes including *Cxcr3* and increases cell surface CXCR3 expression on effector CD4^+^ T cells (18). Up-regulation of CXCR3 expression on CD4^+^Foxp3^+^ T cells also requires T-bet and occurs via an IFN-γ-dependent mechanism (19). To understand the relationship between T-bet and CXCR3 expression during blood-stage malaria, we determined if these markers are co-expressed by CD4^+^ T cell populations in the spleen. CD4^+^ T cells from naïve and infected B6 mice were surface stained for CD4 and CXCR3 followed by intracellular staining for Foxp3 and T-bet, and marker expression was analyzed in gated CD4^+^ cells by flow cytometry. First, we analyzed CXCR3 expression on Foxp3^−^ and Foxp3^+^ cells in the gated T-bet^−^ populations (Figure 3A). We observed small but significant increases in the frequencies of Foxp3^−^T-bet^−^ and Foxp3^+^T-bet^−^ cells that expressed CXCR3 on day 8 p.i. compared to naïve B6 mice with a further significant increase in the frequency of CXCR3^+^ cells in the Foxp3^−^T-bet^−^ population on day 11 p.i. (Figure 3B). We then gated on the T-bet^+^ population and analyzed CXCR3 expression within the two populations (Figure 3A). We observed a dramatic increase in the frequency of cells expressing CXCR3 among Foxp3^−^T-bet^+^ as well as Foxp3^+^T-bet^+^ cells on days 8 and 11 p.i. (Figure 3C). Together, these findings demonstrate that low frequencies of effector CD4^+^Foxp3^−^ T cells and CD4^+^Foxp3^+^ Tregs express CXCR3 in the absence of T-bet expression during *P. chabaudi* AS infection. Importantly, CXCR3 expression was highly up-regulated, regardless of Foxp3 expression, and was co-incident with up-regulated T-bet expression and increased frequency of IFN-γ^+^ effector CD4^+^ T cells on day 8 p.i. as shown above.

### CD4^+^ T Cells From Infected B6 Mice Migrate *in vitro* to C-X-C Chemokines

**Figure 4:**
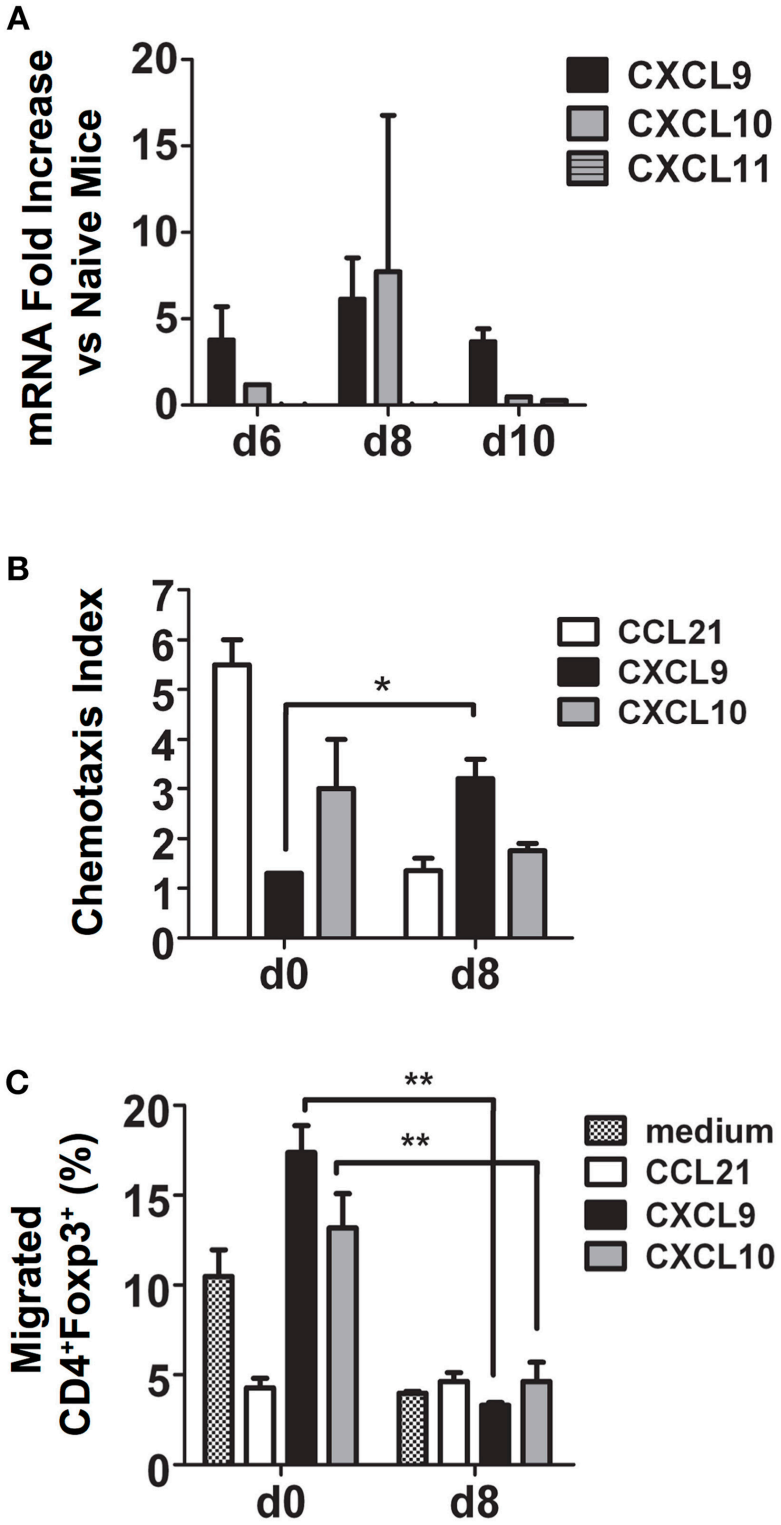
During malaria, CD4^+^CXCR3^+^ T cells migrate to CXCL9 and CXCL10 and are predominately effector CD4^+^Foxp3^−^ T cells. Spleen cells were collected from naïve (d0) and infected B6 mice on the indicated days after i.p. infection with 1 × 10^6^ *P. chabaudi* AS pRBC. **(A)** Total mRNA was extracted and chemokine expression was determined using the RT^2^ Profiler PCR Array for mouse chemokines and receptors. The fold increase in mRNA expression of CXCL9, CXCL10, and CXCL11 in infected vs. naïve mice on days 6, 8, and 10 p.i. is shown. Data are presented as mean ± SEM and are representative of one of two replicate experiments with 3 mice at each time point. **(B)** CD4^+^ T cells were purified from the spleens of naïve (d0) and infected B6 mice on the indicated days p.i., 5 × 10^5^ cells were added to the top chamber of Transwell plates, and their chemotactic response to CCL21, CXCL9, and CXCL10 was determined. Migrated cells in the bottom chamber were collected, counted, and the chemotaxis index was determined as described in the Materials and Methods. Data are presented as mean ± SEM and are representative of one of two replicate experiments with five mice at each time point. ^*^p < 0.05. **(C)**. Migrated cells from the bottom chambers of the Transwell plates described above were collected and surface stained with FITC-conjugated anti-CD4 mAb followed by intracellular staining for Foxp3. The cells were analyzed by flow cytometry to determine Foxp3 expression. The percentages of CD4^+^Foxp3^+^ Tregs that migrated to CCL21, CXCL9, and CXCL10 are shown. Data are presented as mean ± SEM and are representative of one of two replicate experiments with 5 mice at each time point. ^**^p < 0.01.

CXCR3 and its ligands, the IFN-γ-inducible C-X-C chemokines CXCL9, CXCL10, and CXCL11, are important for migration of activated CD4^+^ Th1 cells to inflamed tissues and sites of infection (24). To determine if CXCR3 ligands are induced during *P. chabaudi* AS infection, we examined chemokine mRNA expression in the spleen of B6 mice at various times after infection by microarray. We observed *Cxcl9* and *Cxcl10* but not *Cxcl11* expression was up-regulated in the spleen of infected B6 compared to naïve mice (Figure 4A).

To determine if increased expression of CXCR3 on CD4^+^ T cells during malaria was reflected in their ability to migrate *in vitro* to C-X-C chemokines, CD4^+^ T cells were purified from the spleens of naïve and infected B6 mice on day 8 p.i. and migration to CXCL9 and CXCL10 was assessed using Transwell plates as previously described (19). Medium and the CCR7 ligand CCL21, highly expressed on naïve T cells, were used as negative and positive controls, respectively. As expected, CD4^+^ T cells from naïve B6 mice migrated to CCL21 with a chemotaxis index 5-fold higher than cells from infected mice (Supplementary Figure 2 and Figure 4B). Splenic CD4^+^ T cells from infected B6 mice migrated in response to CXCL9 approximately three times more than cells from naïve mice with a significantly higher chemotaxis index, while no significant difference was observed for migration to CXCL10. To investigate if the difference in CXCR3 expression between effector CD4^+^Foxp3^−^ T cells and CD4^+^Foxp3^+^ Tregs during *P. chabaudi* AS infection described above correlated with differences in migration *in vitro* to CXCR3 ligands, cells were collected from the bottom chamber of the Transwell plate and surface stained for CD4 followed by intracellular staining for Foxp3 and the expression of Foxp3 was determined by flow cytometry. Approximately 13–17% of CD4^+^ T cells from naïve mice that migrated to CXCL9 and CXCL10 were Foxp3^+^ cells while <5% that migrated to CXCL21 were CD4^+^Foxp3^+^ (Figure 4C). Surprisingly, there were significant decreases in the frequency of CD4^+^Foxp3^+^ T cells that migrated to CXCL9 and CXCL10 on day 8 p.i. This was despite significant increases in the number of Tregs that expressed CXCR3 and in the level of CXCR3 expression as described above (Figure 4C). In contrast, more than 80% of CD4^+^Foxp3^−^ T cells from infected mice migrated to the C-X-C chemokines (data not shown). These findings indicate that expression of CXCR3 on effector CD4^+^Foxp3^−^ T cells may be important in trafficking of these cells to the spleen during malaria. In contrast, the ability of CD4^+^Foxp3^+^ Tregs to migrate to CXCR3 chemokines decreased significantly with infection. This observation is consistent with our observation of a higher number of CD4^+^ Foxp3^−^ T cells compared to CD4^+^ Foxp3^+^ Tregs in the infected spleen of B6 mice on day 8 p.i. (6).

### The Course and Outcome of *P. chabaudi* AS Infection in WT vs. CXCR3^−/−^ Mice

**Figure 5:**
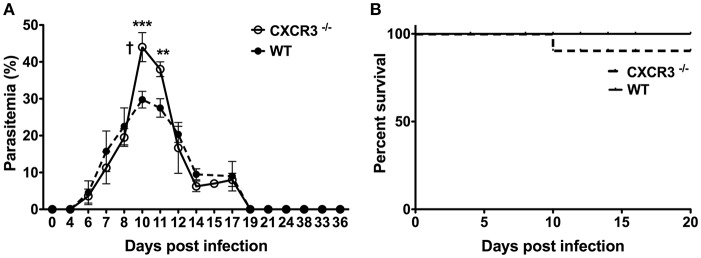
The course and outcome of *P. chabaudi* AS infection in WT and CXCR3^−/−^ mice. WT B6 and CXCR3^−/−^ mice were infected i.p. with 1 × 10^6^ *P. chabaudi* AS pRBC. **(A)** Course of parasitemia in WT and CXCR3^−/−^ mice (*n* = 5 mice/group). Data are presented as mean ± SEM and are representative of one of two replicate experiments. †, indicates one mouse died on day 10 p.i. ^**^p < 0.01; ^***^p < 0.005. **(B)** Cumulative survival of WT and CXCR3^−/−^ mice (*n* = 10 mice per group).

To determine if a deficiency in the chemokine receptor CXCR3 alters the course and outcome of blood-stage malaria, WT B6 and CXCR3^−/−^ mice were infected with *P. chabaudi* AS and the percentage of parasitized RBC in the peripheral blood and survival were determined. Parasitemia increased to similar levels through day 8 p.i. in infected WT and CXCR3^−/−^ mice (Figure 5A). Parasitemia peaked on day 10 p.i. in both genotypes, but the percentage of pRBC was significantly higher in infected CXCR3^−/−^ compared to WT mice. Parasitemia was still significantly higher in CXCR3^−/−^ than WT mice on day 11 p.i., but decreased to levels similar to WT mice by day 12 p.i. WT as well as CXCR3^−/−^ mice cleared the infection by 3 weeks p.i. On day 10 p.i. when parasitemia peaked at high levels, 10% (1/10) of infected CXCR3^−/−^ mice succumbed to infection (Figure 5B). Survival of CXCR3-deficient mice however was not statistically different from the survival of WT mice. WT as well as CXCR3^−/−^ mice were immune to challenge infection (data not shown). Together, these data indicate that the ability to efficiently control parasite replication during acute *P. chabaudi* AS infection was decreased in the absence of CXCR3 but there was little or no effect on survival.

## Discussion

Here, we investigated the contribution of the chemokine receptor CXCR3 to immunity to blood-stage malaria infection. First, we identified the phenotype of CD4^+^ T cells in the spleen that expressed this marker. We observed that the frequency and number of CD4^+^CXCR3^+^ T cells increased dramatically in the spleen of B6 mice during acute *P. chabaudi* AS infection. The timing of the increase coincided with increased CD4^+^ T-bet^+^IFN-γ^+^ T cells in the spleen observed by our laboratory and other investigators (6, 8). We further observed that CXCR3 was up-regulated on effector CD4^+^Foxp3^−^ T cells as well as Foxp3^+^ Tregs, implying these Tregs may be akin to the Th1-like Tregs described by Koch et al (19).

Previously, we observed there are significant increases in the frequency and number of CD4^+^Foxp3^+^ Tregs during acute *P. chabaudi* AS infection in B6 mice followed by a transient decrease until the infection is resolved (6). In the present study, we observed that Foxp3^+^ Tregs expressing T-bet increased significantly. But the increases in both the frequencies and numbers were significantly lower than the responses of effector CD4^+^Foxp3^−^ T cells. Similar to effector CD4^+^ T cells, a high frequency of Foxp3^+^T-bet^+^ Tregs also expressed CXCR3, further supporting the notion that these Tregs are likely Th1-like Tregs.

The findings described in the present manuscript further demonstrate that CD4^+^ T cells from infected B6 mice migrated *in vitro* to the CXCR3 ligand CXCL9 with a significantly greater chemotaxis index than cells from naïve mice. CD4^+^Foxp3^+^ T cells also migrated to CXCR3 ligands, although to a lesser extent than effector CD4^+^ T cells. Interestingly, a significantly greater percentage of Foxp3^+^ Tregs from naïve B6 mice migrated to CXCR3 ligands than cells from infected mice, suggesting the possibility that expression of CXCR3 on Tregs is down-regulated during infection. However, additional experiments are required to address this possibility.

To address the *in vivo* role of CXCR3 during blood-stage malaria, we compared the course and outcome of *P. chabaudi* AS infection in WT (B6) and CXCR3-deficient mice. CXCR3^−/−^ mice had significantly higher parasitemia levels around the time of peak parasitemia than WT mice. There were no significant differences in parasitemia levels during chronic infection and survival was similar in WT and CXCR3^−/−^ mice. It should be noted that the increases in CD4^+^CXCR3^+^ T cells and CD4^+^IFN-γ^+^ T cells observed in the spleen of infected WT mice were coincident with high parasitemia levels in infected CXCR3^−/−^ mice. This suggests a possible role for CXCR3 in controlling parasite replication during acute *P. chabaudi* AS infection. Additional studies however will be required to address the underlying defect in immune responses to blood-stage malaria in CXCR3^−/−^ mice.

Despite studies demonstrating various Th cell subsets are activated during malaria, Th1 and Tfh cells have emerged as essential partners for the development of protective cell-mediated, antibody-dependent immunity to malaria with IFN-γ secreting Th1 cells playing a critical role (2). In addition to CD4^+^ Th1 and Tfh cells, Foxp3^+^ Tregs play an important regulatory role during malaria as a counterbalance to control excessive pro-inflammatory responses and limit immunopathology (4, 6, 7). The proliferation and suppressive activity of Tregs must be restrained during malaria as well as infection with other intracellular parasites to prevent uncontrolled replication of the pathogens (19). *P. chabaudi* AS-resistant B6 mice, used in this study, have a high ratio of effector CD4^+^ T cells to Foxp3^+^ Tregs during acute infection (6, 25). Availability of IL-2, important for the proliferation and survival of Foxp3^+^ T Regs, is crucial for controlling Treg expansion during malaria as shown by increased Foxp3^+^ Tregs and uncontrolled parasite replication in mice infected with *P. chabaudi* AS or *P. yoelii* after treatment with an IL-2-anti-IL-2 mAb complex (6, 7, 26).

To mediate their suppressive effects on effector CD4^+^ T cells, it is critical that Foxp3^+^ Tregs migrate to target tissues where Th1 and Tfh cells have accumulated (27). The spleen is the major site of immune responses to blood-stage malaria and is important for the accumulation of innate immune cells including dendritic cells and NK cells as well as CD4^+^ T cells and B cells involved in adaptive immunity (23). Previously, we observed that CD4^+^Foxp3^+^ Tregs are located almost exclusively in the T cells areas of the white pulp (6). Foxp3^+^ Tregs are known to express various chemokine receptors which direct their migration to sites of Th cell-mediated inflammation (28). Although previous studies showed that CXCR3 expression is up-regulated on splenic CD4^+^ T cells during *P. berghei* ANKA infection, the phenotype of these cells was not defined in detail (15). Furthermore, the significance of the observation of increased CXCR3 expression on splenic CD4^+^ T cells during malaria was unclear.

We observed that CXCR3 expression was associated with the ability of effector CD4^+^ T cells to migrate *in vitro* to the IFN-γ-inducible C-X-C chemokines especially CXCL9. This finding is consistent with the importance of CXCR3 in Th1 cell recruitment to sites of inflammation and intracellular pathogen infection (19). We also observed that Foxp3^+^ Tregs from naïve B6 mice and to a lesser extent from infected mice migrated *in vitro* to CXCL9 and CXCL10. This observation suggests the importance of CXCR3 expression in the trafficking of these cells to the spleen and possibly in regulating their suppressive function during malaria. A CXCR3-dependent mechanism has been shown to be important in Treg recruitment to sites of inflammation in the periphery and central nervous system as well as in an experimental model of liver disease (19, 29, 30). Together, these findings suggest that Foxp3^+^ Tregs, like effector CD4^+^ T cell subsets, respond to local cytokines by modifying their chemokine receptors including CXCR3 to promote their migration to sites of inflammation and infection where they function to suppress immune responses.

The transcription factor T-bet is the master regulator of Th1 cell generation and effector function and is required for IFN-γ secretion (18). T-bet is essential for increased CXCR3 expression by directly transactivating the *Cxcr3* promoter (21, 31). In the absence of T-bet, Th1 cells are unable to migrate *in vivo* to sites of inflammation and fail to migrate *in vitro* in response to the CXCR3 ligands CXCL10 or CXCL11 (21). Similar to conventional CD4^+^ T cells, Foxp3^+^ Treg populations have been identified with transcriptional profiles analogous to their effector Th cell counterparts and critical for their suppressive functions (28). Indeed, T-bet expression in Foxp3^+^ Tregs has emerged as important for regulating Th1-mediated inflammation in various disease models in mice including in Foxp3-deficient Scurfy mice, airway hypersensitivity, autoimmune diabetes, and islet cell allograft rejection (19, 27, 32, 33). Moreover, disease modifying T-bet^+^Foxp3^+^ Tregs also express CXCR3 which facilitate their migration to sites of Type 1 inflammatory responses. Th1-like Foxp3^+^ Tregs have been shown to produce IFN-γ and to express high levels of inhibitory molecules such as CTLA-4 as well as high IL-10 and TGFβ mRNA levels (19, 32, 34). Foxp3^+^ Tregs expressing T-bet and CXCR3 have also been described in humans (35).

T-bet^+^Foxp3^+^ Tregs have been observed in mice infected with *Mycobacterium tuberculosis* and *Toxoplasma gondii* suggesting these cells may be important in infections with intracellular pathogens that induce highly polarized Th1 responses (19, 34, 36). In the present study, we observed that Foxp3^+^ Tregs that co-express T-bet and CXCR3 increase significantly in the spleens of B6 mice during acute *P. chabaudi* AS infection. Recent studies indicate T-bet^+^ Tregs increase during acute *P. vivax* infection while the frequency of Foxp3^+^ Tregs that express T-bet is stable in patients after treatment (20). The exact role of T-bet^+^Foxp3^+^ Tregs in suppressing effector CD4^+^ T cell responses during malaria has not yet been resolved.

In conclusion, we have identified a population of CD4^+^Foxp3^+^ Tregs that co-express CXCR3 and T-bet in the spleen during acute *P. chabaudi* AS infection. Altogether, our findings indicate that CXCR3 expression on effector CD4^+^T-bet^+^Foxp3^−^ cells may contribute to the migration of these cells to the spleen during malaria suggesting a role for CXCR3 in immunity to *P. chabaudi* AS infection.

## Data Availability

The raw data supporting the conclusions of this manuscript will be made available by the authors, without undue reservation, to any qualified researcher.

## Ethics Statement

Mice were maintained and handled according to the guidelines of the Canadian Council on Animal Care and the McGill University Animal Care Committee (ACC). All procedures on experimental mice were approved by the McGill University ACC (Protocol #2015-3750).

## Author Contributions

FB, first author, designed and executed all the experiments, performed data analyses, prepared drafts of graphics for all relevant data, and was responsible for writing of first draft of the manuscript. CP, second author, co-supervised Dr. Berretta on the project, provided scientific input in the conception of some experiments, and contributed to the editing of the final manuscript. MS, senior author, is the Director of the laboratory and primary supervisor of Dr. Berretta. She provided scientific input in experimental design and to ensure that the data were soundly interrogated and interpreted. She was responsible for the final editing of the manuscript.

### Conflict of Interest Statement

The authors declare that the research was conducted in the absence of any commercial or financial relationships that could be construed as a potential conflict of interest.
