# Supplementary figures and images for: Plasmodium chabaudi AS Infection Induces CD4+ Th1 Cells and Foxp3+T-bet+ Regulatory T Cells That Express CXCR3 and Migrate to CXCR3 Ligands
Source: Front Immunol. 2019 Mar 11;10:425. doi: 10.3389/fimmu.2019.00425 (PMC6422055; doi:10.3389/fimmu.2019.00425)

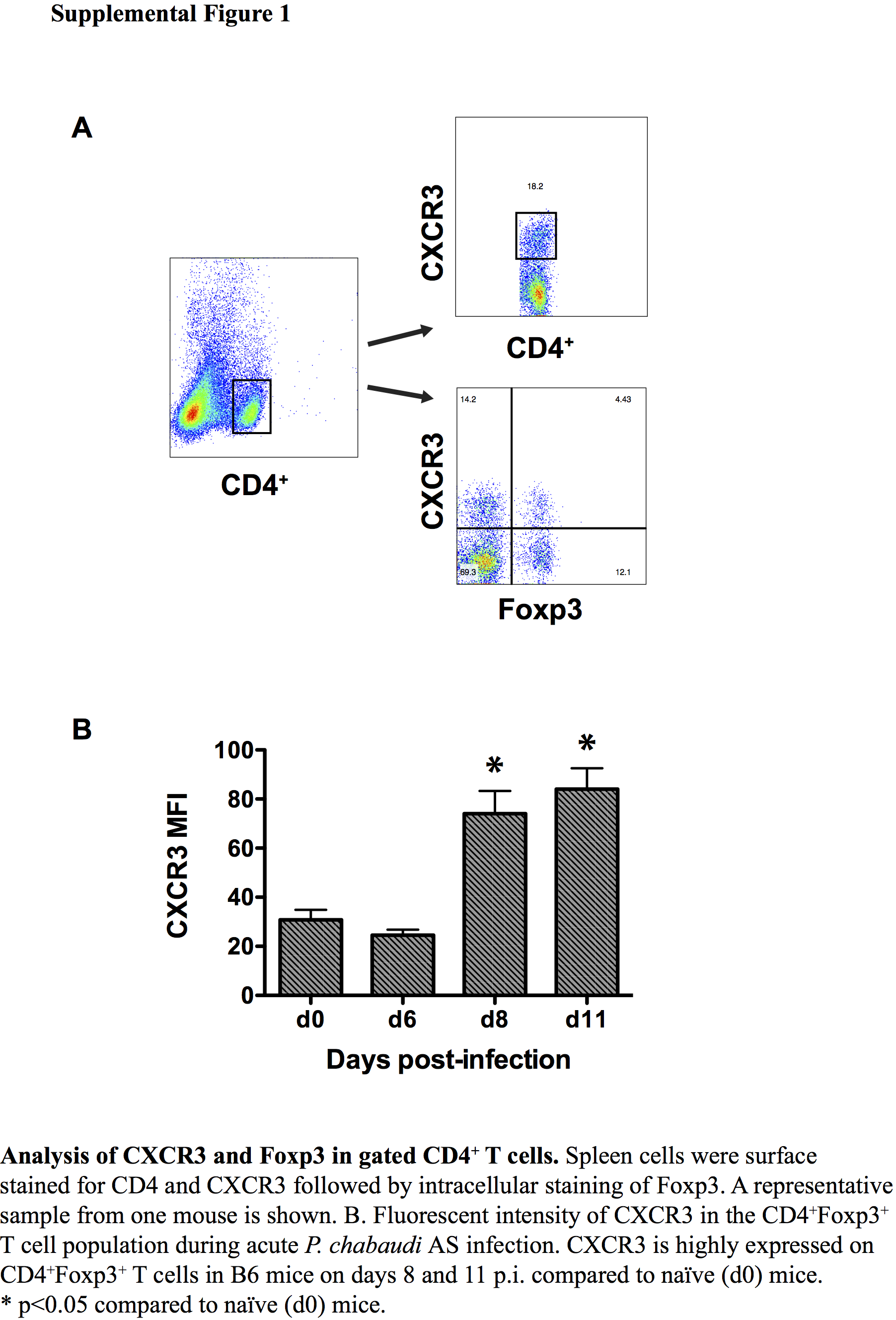

Supplement: Supplementary file 1 [file Image_1.TIFF]

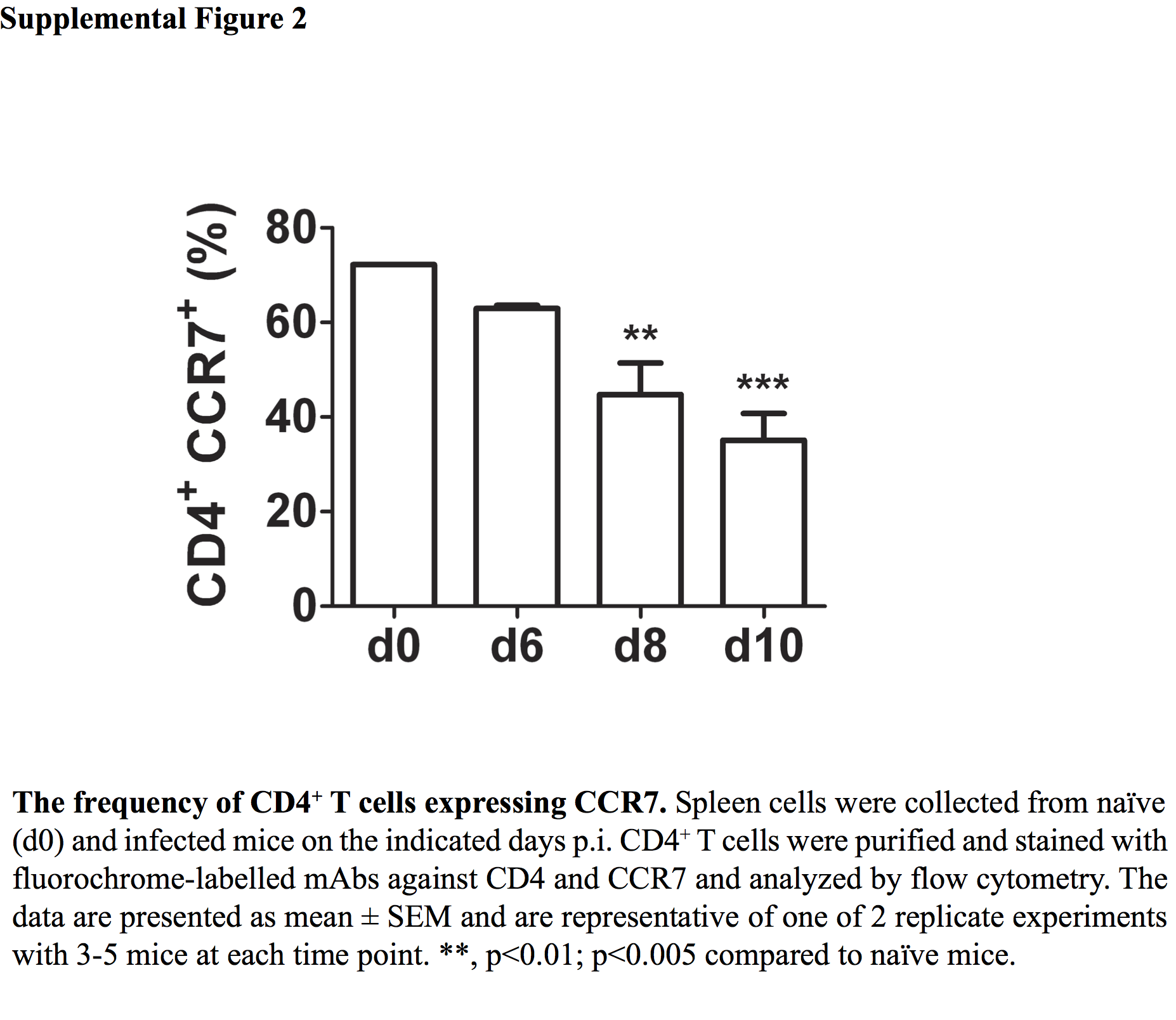

Supplement: Supplementary file 2 [file Image_2.TIFF]
